# Texturing Technologies for Plastics Injection Molding: A Review

**DOI:** 10.3390/mi13081211

**Published:** 2022-07-29

**Authors:** Davide Masato, Leonardo Piccolo, Giovanni Lucchetta, Marco Sorgato

**Affiliations:** 1 Department of Plastics Engineering, University of Massachusetts Lowell, Lowell, MA 01854, USA; 2 Department of Industrial Engineering, University of Padova, 35131 Padova, Italy; leonardo.piccolo@unipd.it (L.P.); giovanni.lucchetta@unipd.it (G.L.); marco.sorgato@unipd.it (M.S.)

**Keywords:** surface texturing, surface engineering, plastics manufacturing, injection molding, micro-manufacturing

## Abstract

Texturing is an engineering technology that can be used to enable surface functionalization in the plastics injection molding industry. A texture is defined as the geometrical modification of the topography by addition of surface features that are characterized by a smaller scale than the overall surface dimensions. Texturing is added to products to create novel functionalities of plastic products and tools, which can be exploited to modify interactions with other materials in contact with the surface. The geometry, dimensions, and positioning on the surface define the function of a texture and its properties. This work reviews and discuss the wide range of texturing technologies available in the industry. The advantages and limitations of each technology are presented to support the development of new surface engineering applications in the plastics manufacturing industry.

## 1. Introduction

The broad range of properties that characterize polymers have made texturing a significant segment for the plastics industry. The value of a textured plastic part can be very high thanks to its added functionalities. Consumer products with surface textures are widespread, offering users a wide array of looks and feels. The texturing of plastic parts finds applications for both consumer and high-end engineering products. The functionalities introduced by surface texturing range from simple aesthetics (e.g., rigid plastic packaging) to advanced biomedical applications (e.g., scaffolds for tissue engineering). More recently, texturing has been used to functionalize injection molds to improve both the process’s filling and ejection phases.

Texturing plastic parts by replicating a micro- or nano-structured tool has been demonstrated as a cost-effective solution to generate different patterns on three-dimensional surfaces. Texture replication requires the consideration of design, material, and processing factors [1]. However, the extensive industrial polymer processing knowledge has constituted an excellent basis for developing the technology. The most common technologies used to replicate micro- and nano-structured technologies include injection molding [2,3], injection-compression molding [4], hot-embossing [5,6], roll-to-roll [7], and other variants of these technologies. The understanding of replication during processing requires the evaluation of polymer properties, processing parameters, and product functionality. Several researchers have studied extensively how different parameters affect replication for different processes.

**Figure 1:**
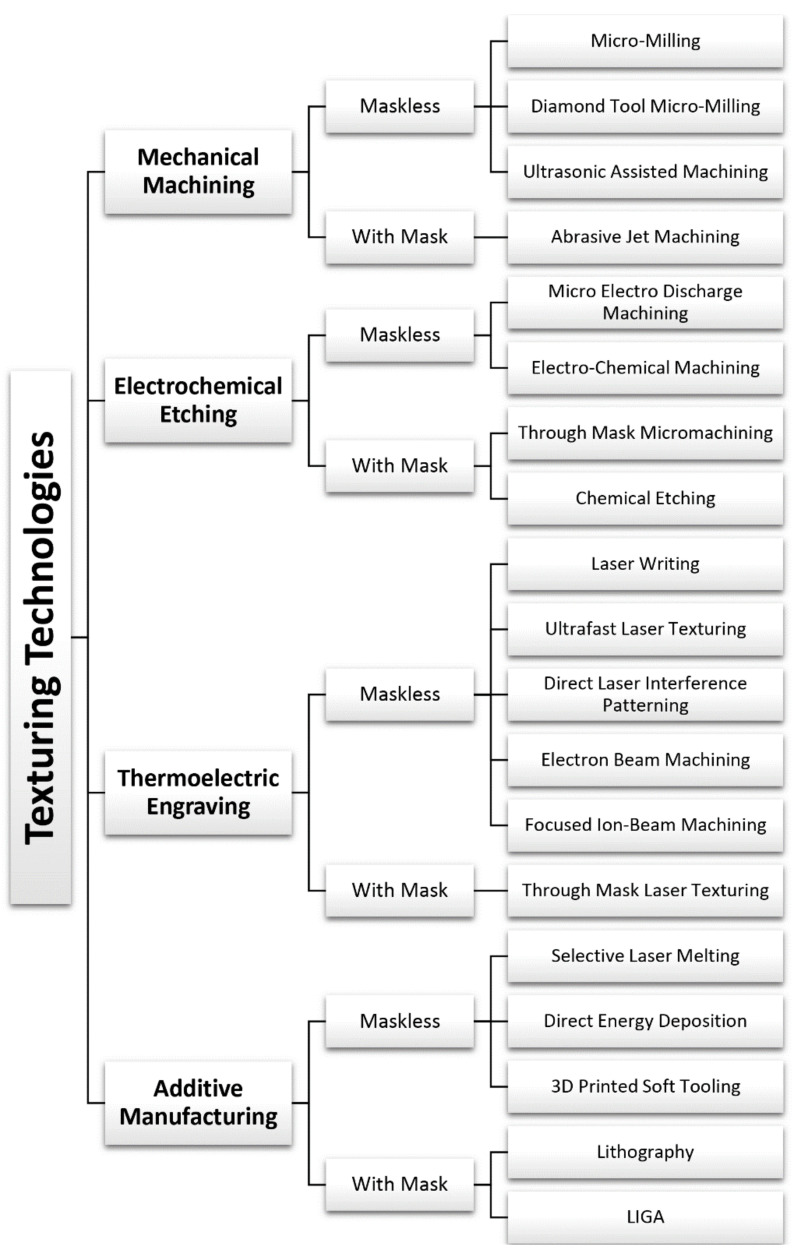
Summary and classification of texturing processes.

This review presents the most common texturing technologies available for plastics manufacturers. The different technologies are discussed and compared to highlight their advantages and disadvantages. The different texturing techniques exploit diverse types of energies to generate a specific surface topography. Texturing processes can be classified as mechanical, thermo-electric, electro-chemical, and additive, depending on the primary working principle. The substrate materials allowed by each technology is briefly discussed for each technology. Each one of these processes can be further divided into processes that exploit a mask to work on the surface selectively or processes that concentrate the energy on a small spot of the surface (cf. Figure 1).

## 2. Mechanical Machining

### 2.1. Micro-Milling

Micro-milling is a mechanical machining process that removes material from the workpiece, exploiting the energy of the relative of the tool. Micro-milling is defined based on the cutting tool’s dimensions, which should be smaller than 1 mm. Micro-milling has been widely used to create textures on different materials for different applications [8,9]. Micro-milling is typically scaled down from the conventional milling process; however, many issues must be considered.

Texturing by micro-milling can involve long machining times, depending on the pattern geometry, dimensions, and properties of the workpiece material. This process can produce 3D shapes with features as small as few tenths of microns [10]. The most advanced computer-controlled milling machine provides the precise and simultaneous movement of many axes, enabling the creation of complicated tool paths for high flexibility to the texture structure shape. The CNC control allows to machine complex structures with high accuracy and precision. However, the reduced diameter and the need for mechanical stability during cutting leads to limitations for the tool length. In general, the small tool dimension limits the process’s ability to texture areas that are difficult to reach. Micro-milling tools can be classified into four main types, depending on their cutting-edge shape. These types are flat-end mill, ball-end mill, tapered ball-end mill, and bull nose mill.

Machining at the micro-scale presents several challenges that cannot be addressed by traditional milling approaches. Therefore, micro-milling cannot be considered as a downscaling of the traditional process. A micro-milling process is challenging to control, and several processing factors have to be considered [11]. The machine, tool, and toolpath have to be redesigned to promote precise and effective tool–workpiece contact [12]. In particular, the workpiece properties, the tool geometry, and the tool materials have to be carefully selected according to the process speeds.

Micro-milling often uses high-speed machining techniques with spindle rotations between 40,000 and 50,000 rpm and specialized CAM programming that requires specifically designed machines. Along with a rigid structure, the spindle must be stable to minimize tool vibration and prevent thermal expansion. Indeed, any vibration at the tooltip deteriorates the surface quality and shape accuracy. Vibrations are amplified as the cutting force increases and as the tool diameter is reduced [13].

When texturing the workpiece surface by micro-milling, the cutter’s size is comparable to the chip thickness and the material grain size [14]. Downscaling milling technologies and approaches do not lead to good texture quality and the mechanical resistance of the micro-milled texture. The presence of large grains on the workpiece material adversely affects the quality of the process, causes the spring break effect that increases the obtained surface roughness. The tool shape and the cutting tool’s edge must be considered not only in regard to the designed features but also to the grain size. Coated tools are commonly exploited for milling such small features to enhance tool performance. However, the tool dimensions are usually larger.

The behavior of tools employed in micro-milling is hard to predict as chip clogging may easily result in a catastrophic failure, and fatigue failure may occur because of the high spindle speeds. The most common processing issue is burr formation, which is usually not acceptable, mainly because the deburring of such small features is challenging and often not feasible. Typically, the burr formation is minimized by selecting the optimal cutting parameters and tool material and shape. Compared to conventional cutting tools, micro-tools wear rapidly due to their low rigidity, leading to higher force variation and earlier tool failure. Due to this rapid tool wear and the higher fluctuation of cutting forces, the micro-feature part’s dimensional accuracy deteriorates [15]. Tool wear is highly affected by spindle speed, feed per tooth, cutting depth, lubrication, and workpiece material [14].

### 2.2. Diamond Tool Micro-Milling

Diamond tools are used for micro-milling applications that demand high accuracy. The process can generate a surface roughness of only a few nanometers and features as small as some microns. All the advantages of having multiple degrees of freedom during machining are shared with conventional micro-milling for a wide variety of machinable 3D structures [16]. Diamond tools have highly desirable properties, such as low friction, high wear resistance, and maintaining a sharp cutting edge. Single crystal diamonds can be used for selected applications, but they are typically replaced by polycrystalline diamond (PCD) tools (also known as compacts). The presence of randomly oriented crystals hinders crack propagation and improves the tool toughness. The shape and sharpness of the tool have to be correctly designed to reduce the diamonds’ brittle behavior. Tool wear may occur through microchipping due to thermal stresses and oxidation and through transformation to carbon caused by the heat generated during cutting [17]. The tool’s alignment to the workpiece and machine axis is of vital importance to the dimensional accuracy and tool life. A deviation of only a few micrometers can lead to deformed structures and early tool wear. Therefore, the generation of an optimized tool path is crucial for diamond tool milling.

### 2.3. Ultrasonic-Assisted Machining

Ultrasonic-assisted machining is used for texturing by applying high-frequency vibration to either the workpiece or the cutting tool. The tool can be a turning tool, a milling cutter, or a grinding wheel [18]. The vibration wave is characterized by frequencies higher than 20 kHz and amplitudes between 1–10^1^ μm [19]. The integration of ultrasonic vibration with a conventional machining process allows the generation of different topographies than a non-vibrating process. The relative tool–workpiece vibration induces micro-scale modification to the machining toolpath, resulting in more complex textures on the workpiece’s surface.

**Figure 2:**
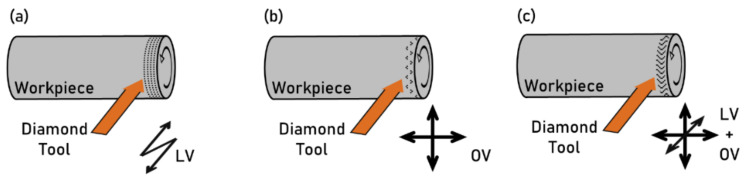
Different vibrational modes for the ultrasonic assisted turning process. (**a**) longitudinal vibration (LV); (**b**) orthogonal vibration (OV); (**c**) LV and OV applied together.

The ultrasonic vibration can be integrated with processes such as grinding, milling, turning, and cutting. In general, the vibrating device is attached to the cutting tool allowing ease of installation and control. Two types of vibration modes can be applied to the tool to machine different textures. The Longitudinal Vibration (LV) mode is the vibration along the direction of the depth-of-cut. In the LV mode, the conventional machining process is transformed into an intermittent cutting operation, enabling micro-dimples realization (Figure 2a). The Orthogonal Vibration (OV) mode makes the tool vibrate in the plane orthogonal to the depth-of-cut, controlling the cutting tool’s micro-path along the surface. Compared to the LV mode, a one-dimensional vibration, the OV mode allows tool vibration along two different directions (Figure 2b). An elliptical vibration can be generated to achieve more complex textures by modulating the amplitudes and the frequencies of the oscillations along the two dimensions. The two modes can be applied together to make a three-dimensional vibration (Figure 2c) [20].

The vibrating device is realized using one or more piezoelectric plates assembled between cylindrical ultrasound horns. The high-frequency sinusoidal voltage excites the piezoelectric, which expands and contracts, generating a vibration of the tool. Different types of vibrating devices are realized using multiple piezoelectric plates of different shapes. For axis-symmetric tools, a one-dimensional vibrator can be built with one plate to achieve an LV mode. The OV mode can be obtained by stacking different plates. By feeding the different vibrators with selected electrical signals, it is possible to generate different vibration modes. For example, a circular or elliptical vibration can be obtained by selecting the amplitudes of the signals. The two vibrators can be connected in series, so that the cutting tool gains three vibrational degrees of freedom.

The dimension and characteristics of the texture generated by ultrasonic-assisted machining depend on the tool geometry, on the combination of vibration mode, and on the feed path. In this process, the geometry of the texture is easier to control when the shape of the tool is simple. For example, diverse textures can be achieved by diamond grinding when controlling the tool vibration. However, the irregular geometrical shapes of grinding wheels do not allow control over the generated topography. The tool–workpiece interaction resulting from this process is too complicated, and the tool’s wear is difficult to predict. General guidelines such as increasing the tool hardness and decreasing the depth of cut are helpful to limit the high wear driven by the high-frequency tool–workpiece contact.

### 2.4. Abrasive Jet Machining

**Figure 3:**
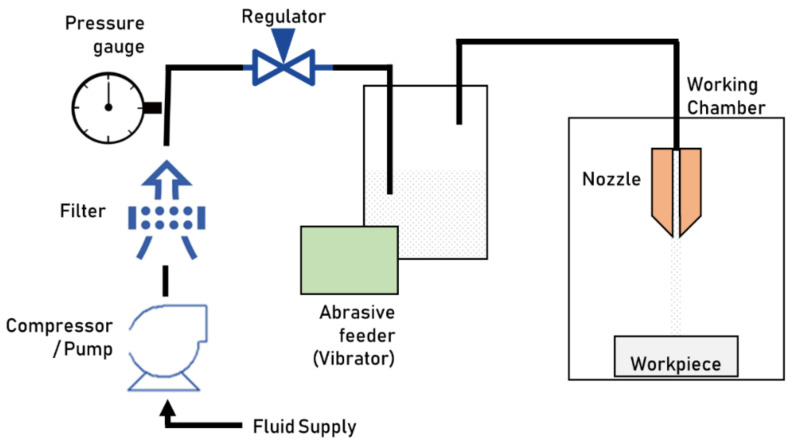
Schematics of the abrasive jet machining setup.

Abrasive jet machining exploits abrasive particles’ kinetic energy to remove material from the workpiece creating a texture. Typically, aluminum oxide, silicon carbide, or glass beads, are used as abrasive particles. The particle size greatly affects the Material Removal Rate (MRR) by controlling the kinetic energy [21]. Typical particle sizes range from 0.25 to 60 µm. The abrasive particles are controlled using a fluid, usually air (for Abrasive Air Jet Machining, AAJM) or water (Abrasive Water Jet Machining, AWJM). The fluid is compressed or pumped, propelling the abrasive particles through a nozzle. The abrasive feeder supplies the required abrasive particles (Figure 3). The fluid–abrasive mixture (called slurry when using water) is accelerated by flowing through the nozzle and strikes the workpiece surface, chipping and eroding microparticles [8]. The fluid also cools the workpiece down, removes the stagnant abrasive, and clears the debris from the working area.

Depending on the desired geometry, roughness, and integrity of the machined features, many variations of Abrasive Jet Machining (AJM) have been developed. The higher jet viscosity in AWJM leads to many advantages over AAJM, such as better roughness, higher accuracy, more homogeneous cooling, and the better cleaning of the workpiece.

Dispersing ferromagnetic particles in the slurry further enhances the jet’s viscosity and stability in Magnetorheological Polishing (MRP). The jet, stabilized by magnets, is 20 times more stable than the conventional slurry jet. This process is implemented for geometries that are difficult to reach with the jet. Cryogenic-assisted abrasive jet machining is exploited when machining low-hardness materials. The Material Removal Rate (MRR) of the process is enhanced by heating the workpiece in thermally assisted abrasive jet machining.

These processes are applied for micro mold-die texturing applications with feature dimensions down to 10 µm-wide trenches [22] or textures with pockets as big as 50 microns [23]. The texturing process is conducted by masking the workpiece surface with an erosion-resistant material. The mask itself defines the machinable size since the material needs to withstand the erosion, and thus needs sufficient strength. Moreover, machining smaller features require smaller abrasive particles that have less kinetic energy due to the low mass, resulting in a different material removal mechanism (i.e., plastic removal mechanism). These conditions lead to lousy selectivity between the mask and the substrate, making it difficult to machine 100 µm channels with an aspect ratio deeper than 2.5 [21].

## 3. Electrochemical Etching

Etching uses energy (i.e., chemical, electrical, thermal) to remove material from the workpiece’s surface, creating a texture. In an etching process, the workpiece is not affected by any mechanical contact. The texture is generated by masking selected areas and by etching the unmasked portions of the surface. Different etching processes have been developed depending on the type of energy being used and the masking technique. Chemical, electrical, ion, or combination of these energies can break the bonds of thin layers of the exposed portion of a metal surface. The masking material should resist the etching unless the energy could be focused only on selected areas. The substrate material should be selected accordingly to the characteristics of the etching method, as some technologies are limited to certain subset materials.

The resolution of the features that compose the texture depends strongly on the method used to mask the surface. Depending on the printing method, texturing can also be applied to surfaces with irregular shapes. Additionally, few restrictions are related to the shape and distribution of the features that compose the surface texture [24].

### 3.1. Chemical Etching

**Figure 4:**
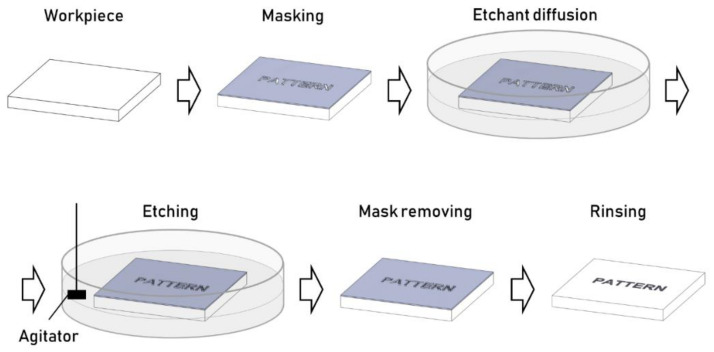
Process steps for a typical chemical etching process.

**Figure 5:**
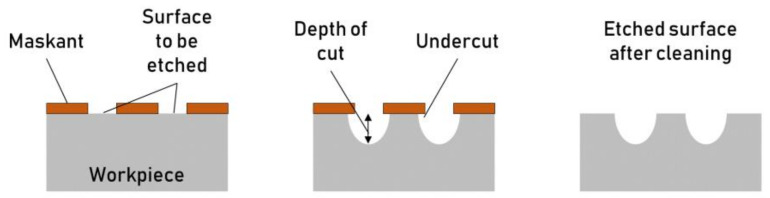
Bi-directional material removal, downward—depth of cut—and laterally—undercut—from the exposed surface.

Chemical etching, also known as wet etching or liquid etching, exploits chemical energy for material removal. The etchant solution is selected according to the material whose surface is being textured. The mask is attached to the surface, defining the geometry of the texture. A chemical etching process involves the following steps (Figure 4):

1. Workpiece preparation and cleaning with an alkaline solution and water. Chemical cleaning ensures proper adhesion of the maskant layer on the metal surface. The solvent selection for the chemical cleaning depends on the maskant kind, the workpiece material, the workpiece surface finish, and the required texture depth. A proper surface cleaning prevents the debonding of the mask, which will result in stray etching.
2. Masking of the workpiece to selectively cover specific areas of the surface. Different masking techniques are available depending on the workpiece’s dimension, the characteristic texture dimension, the required accuracy, and the etching depth. Cut and peel off consists of applying an even coating and peeling off the marked areas. Photochemical masking exploits a photoresist coating that is selectively developed to mask and free certain areas and jet printing techniques, to name a few. As the texture features dimensions shrink down to the microscale, photochemical masking techniques are preferred (Zhang et al., 2016). This process guarantees a high texture shape accuracy without being substantially affected by the pattern complexity. The maskant material has to bond tightly to the workpiece surface and has to be resistant to the etchant.
3. Immersion of the workpiece in the etchant bath. Besides the workpiece and maskant materials, the etchant selection is usually driven by process factors, such as surface finish and material removal rate and etchant availability, cost, and sustainability.
4. Etching of the unmasked areas. The etchant bath can be agitated and heated to increase the material removal rate and uniformity. The material removal rate is typically low and develops in two directions, downward—depth of cut—and laterally—undercut—from the exposed surface (Figure 5). The ratio between the undercut and the depth of cut is known as the “etch factor.” The etch parameter’s control is challenging and requires a deep understanding of the process phenomena, especially when etching texture with small features.
5. Workpiece chemical rinsing to remove the mask layer and etchant residues. At the end of the process, the etchant is cleaned, and the mask has to be removed.
6. Final workpiece rinsing. Finally, as the mask is removed, a final rinsing step with clear water is carried out to remove any chemical used for etching or mask removal.

### 3.2. Electro-Chemical Machining

In Electro-Chemical Machining (ECM), the electrical energy is used to induce a chemical reaction on the workpiece’s surface. The process exploits the principle of Faraday’s laws of electrolysis. ECM follows a procedure similar to a chemical etching process, but the chemical bath is substituted by an electrolytic cell. A small electric DC (Direct Current) potential (5–25 V) is applied between the tool (cathode) and the workpiece (anode) immersed in an electrolyte. The electrolyte usually consists of acid or basic salts diluted in a water solution. The electric current generates the transfer of ions between the electrodes and the workpiece. The metal surface (anode) is textured by removing atoms, which are released into the solution as hydroxyl ions, thus forming metal hydroxides. These hydroxides, insoluble in water, quickly precipitate. The electrolyte solution is flown between the tool and the workpiece to remove the reaction products, allowing higher texturing rates. Electrolyte properties, such as pH, temperature, purity, and hydroxide concentration, are kept uniform using filters and large settling tanks [19].

**Figure 6:**
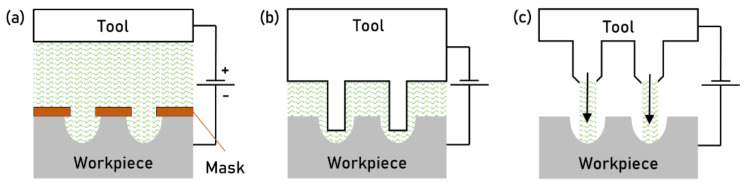
(**a**) Through mask electrochemical machining (**b**) maskless electro chemical machining (**c**) jet ECMM.

Surface texturing is realized by masking the workpiece with a non-conductive material (Figure 6a). This process is also known as Through Mask Electrochemical Machining (TMEM). Alternatively, the texture can be etched on the workpiece surface using a pre-patterned cathode. This process is also known as Maskless Electro Chemical Machining (MECM). The MRR (material removal rate) is mainly affected by the current density; hence a smaller distance between the workpiece and the tool results in a higher MRR (Figure 6b). Avoiding the masking step in MECM makes setup and processing easier. However, the pre-patterned electrode requires a higher investment. When many parts have to be textured with the same topography, MECM is preferred even because the cathode is not affected by wear and can accurately machine the same texture over time [8].

In general, the machined textures’ accuracy is a function of tool or mask design, process parameters such as voltage and electrolyte purity, material clearness (hard spots, inclusions, and sand present some practical difficulties), and complexity of the desired structure shape. The ECM process allows texturing at the microscale for both TMEM and MECM. TMEM is at least a two-step process, requiring accurate surface masking. Depending on the masking technology, free-form surfaces can be accurately masked and textured. Structures with characteristic dimensions below the micron are possible. Masking often determines the major costs of the texturing process. Avoiding the masking step, MECM results in a faster and less expensive process. However, there are substantial limitations on the workpiece surface shape, which are typically flat or cylindrical. Process variations have been developed to enhance surface finish or achieve a higher aspect ratio (i.e., the ratio between the depth and the width of a dimple). For instance, ultrasonic vibration-assisted ECMM (Electro Chemical Micro Machining) and jet ECMM (Figure 6c) are established variants that enhance the capabilities of conventional ECM [25].

The absence of direct contact between the tool and the workpiece, and the minimum heat generated by the electrochemical reaction, results in a workpiece surface without any thermal stress. The process can be automated, leading to processing efficiency and competitive machining time.

### 3.3. Electric Discharge Machining

Electric Discharge Machining (EDM) is used to texture metal surfaces exploiting electrical energy to etch the workpiece. A high potential is induced between the tool and the workpiece to generate a spark. Whenever sparking takes place between two electrodes, a small amount of material is removed from both sides. Multiple sparks are used to machine the material generating the texture. In the EDM process, texturing is achieved either using a pre-textured tool electrode or moving a flat electrode along the workpiece surface. The high potential is created by a capacitor charged from a direct current source. As the capacitor’s relaxation circuit (known as RC circuit) reaches the breakdown voltage, sparking takes place at the point of least electrical resistance, which typically is the smallest inter-electrode gap (IEG). The tool and the workpiece are submerged in a dielectric promoting the localization of the spark energy, flushing the debris away, and cooling the electrodes.

The localized heat generated by the spark removes the material on the workpiece and the tool. The dielectric fluid and the debris remove the heat. The thermoelectric process is characterized by a short cycle time (the charge–discharge time is a few microseconds) and a frequency as high as thousands of sparks per second [26]. The high energy concentrated in a small area dramatically increases the local temperature causing melting and vaporization of the material from both the electrodes. The generated heat vaporizes the dielectric present in the interested area, increasing the local temperature (from 800 up to 20,000 °C) and pressure (i.e., hundreds of atm). The high pressure prevents the electrode from vaporizing. As the pulse is over, the pressure drops, and the superheated electrode material vaporizes. The heat-affected zone is formed directly under the machined surface because of the high temperatures reached during the process. The dielectric fluid increases the dielectric strength, allowing the system to remain non-conductive until the breakdown voltage is reached. Moreover, it promotes minimum ionization time once the breakdown voltage is reached (i.e., ignition delay time) and allows for deionization after the spark has occurred.

**Figure 7:**
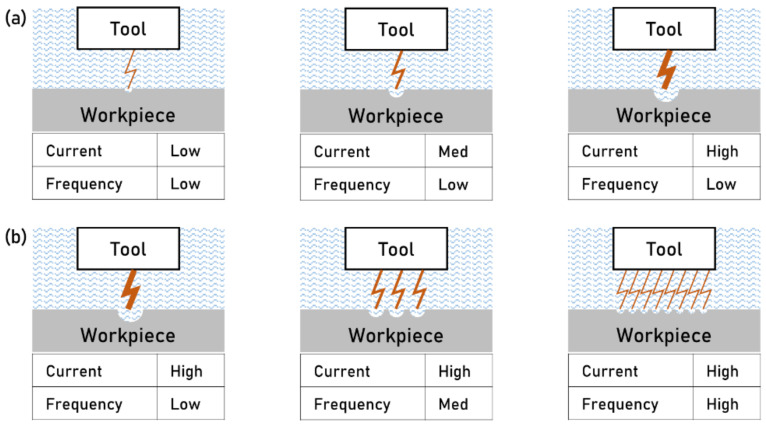
(**a**) Effect of current during sparking on surface finish, and (**b**) effect of frequency during sparking on surface finish.

The material removal forms a crater on the spark’s area, exposing a new smallest IEG to the upcoming spark. The sequence of multiple sparks results in the desired machined texture, determined from tool geometry and processing considerations. The amount of material eroded by every spark and the resulting surface finish both depend on the spark energy, duration, and frequency. The volume of material removed per discharge is typically in the range of 10^−6^–10^−4^ mm^3^. Typical MRR for the process is between 2 and 400 mm^3^/min, depending on geometrical and machining parameters. In general, higher currents lead to larger craters, thus higher MRR but coarser surface finish (Figure 7a). Conversely, higher frequencies divide the energy into many sparks, improving the surface finish (Figure 7b) [27].

The material removal happens on both the tool and the workpiece. However, a good selection of the tool material and the process parameters can limit the tool wear. The choice of tool material is usually driven by properties such as machinability, low wear rate, excellent electric properties, heat conductivity, cost, and availability [28]. Graphite and copper electrodes are widely adopted because they are easy to machine by micro-milling.

The texture accuracy is related to the tool geometry and its wear, which are affected by machining. For this reason, different tools and process parameters are used for the roughing and the finishing phase, allowing a reduction of tool wear. The geometry of the finishing tool has to match the required texture shape. Particular attention should be directed to corners, where the electric field is stronger, leading to an undesired rounding effect [29].

Flushing removes heat and debris from the IEG, thus avoiding damaging arcs in the gap. Various techniques have been developed to clear the gap between the electrodes, such as jet flushing, through flushing electrodes, alternating flushing, and ultrasonic tool vibration. The dielectric fluid bath is circulated using filters and coolers to remove debris and heat. Ineffective flushing causes low MRR, reduced geometrical accuracy, and worst surface finish.

The smaller the texture generated using EDM, the higher accuracy is required for process control. More stable pulse generators (e.g., relaxation type generators) are employed to achieve precise energy output at a short pulse duration. The smaller energies involved and an accurate gap control system allow a substantial reduction in IEG, lowering the breakdown voltage and enabling better dimensional accuracy for the micro-machined features. Moreover, for micro-scale features, tungsten is used for the tool, for its high melting point and tensile strength. However, copper and tungsten carbide were also successfully used.

The minimum size for an EDM-machined texture is in the range of units of micrometers. However, to reach such accuracy and resolution, the tools and the setup should be appropriately designed and manufactured. Moreover, the smaller the texture, the lower the MRR [29].

## 4. Thermoelectric Engraving

### 4.1. Laser Texturing

Laser texturing exploits electromagnetic energy to melt and vaporize the material on the surface, creating a texture. The LASER (Light Amplification by Stimulated Emission of Radiation) source generates a monochromatic and coherent light beam (i.e., light waves with the same wavelength and phase). The laser beam typically shows low divergence (i.e., it can travel for long distances), thus allowing relatively long optical paths. The Laser light properties allow light focusing on small spots with high irradiance, which is decisive for engraving operations.

The wavelength of commonly used laser systems is between 0.2 and 11 µm (Ruby = 0.7 µm, Nd:YAG = 1.0 µm, CO = 2.7 µm, CO2 = 10.6 µm, and Ti:Sapphire = 0.78 µm). Depending on the gain medium being used, two types of lasers can be found: solid-state lasers and gas lasers. Typically, gas lasers can reach higher powers. Indeed, industrial lasers can also be divided into continuous-wave lasers and pulsed lasers. Continuous-wave lasers are used for welding or surface hardening, which requires an uninterrupted supply of energy for a full thermal process. Pulsed lasers are preferable for machining purposes because the controlled pulse energy can enhance the MRR containing the extension of the Heat-Affected Zone (HAZ). Shorter pulse durations lead to smaller HAZ, but typically lasers with short pulse duration are substantially more expensive. The successful formation of a texture requires the evaluation of the interactions between the substrate material and the laser. Laser processes are mostly established for conventional mold materials, such as steel and aluminum. However, the texturing processes for a specific material require application-specific optimization of the process parameters [30].

#### 4.1.1. Laser Writing

Laser writing uses the entire laser beam spot size to ablate and texture the surface of the workpiece. The light irradiation is focused on the metal surface, melting and vaporizing the metal in controlled areas. The heat generated by the laser beam creates a Heat-Affected Zone (HAZ), which could have micro-cracks or nano-bubbles, depending on the processing parameters. The minimum texture size that can be machined depends on the laser beam divergence, which is a function of the laser light quality. For some applications, a laser spot diameter—and consequently feature dimensions—of some tens of micrometers can be machined [31]. In a laser writing process, either the laser source or the workpiece can be moved using a CNC system to texture a complex surface. Typically, the industrial machining systems combine beam control and workpiece control to maximize the texturing area and the accessibility of the laser beam into the workpiece surfaces. Indeed, for complex three-dimensional geometries, texturing might not be possible for areas that are not optically accessible, or for areas on which the laser beam cannot be properly focused.

Fiber laser systems exploit a glass fiber to deliver the laser light from the resonant chamber to the optical head. This laser allows for smaller and more compact laser equipment, which guarantees increased flexibility for moving the optical head around the workpiece. When using a fiber laser, the workpiece is set in the moving stage to increase the machining degrees of freedom. The laser beam’s high stability and flexible configuration allow texturing of complex geometries with good accuracy and precision. High-aspect ratio dimples (i.e., blind holes) are the most common texture machined by laser writing. However, many different textures can be produced, with the main limitation being the minimum laser spot size. The texturing speed is limited by the need to ablate individual features using the laser beam directly.

#### 4.1.2. Through Mask Laser Texturing

**Figure 8:**
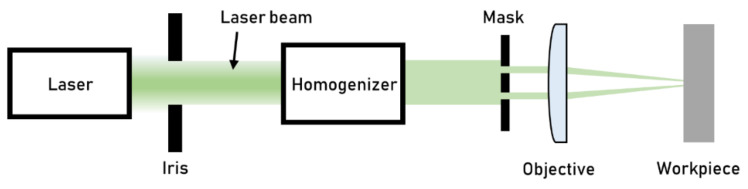
Working principle of through mask laser texturing.

Through mask laser texturing guarantees higher productivity than direct writing by interposing a mask between the laser beam and the focus region. Along the laser path, the irradiance (power per unit area) or the fluence (energy for units of area) is weaker than in the focus area; thus, the mask is not damaged by the laser. The beam is homogenized before hitting the mask to flatten the laser beam’s Gaussian energy profile, allowing for homogenous texturing (Figure 8). The laser beam’s low divergence allows for the accurate projection of the shapes of the mask onto the workpiece surface. Texturing is obtained by the selective irradiation of the workpiece surface, which results in selective ablation [32].

Patterns characterized by various shapes can be engraved with this technique. However, limitations on the minimum size achievable should be taken into consideration. Indeed, the minimum size of the structures has to be higher than the laser light wavelength to avoid diffraction effects. In general, features as big as few microns can be machined using through mask laser texturing.

#### 4.1.3. Ultrafast Laser Texturing

**Figure 9:**
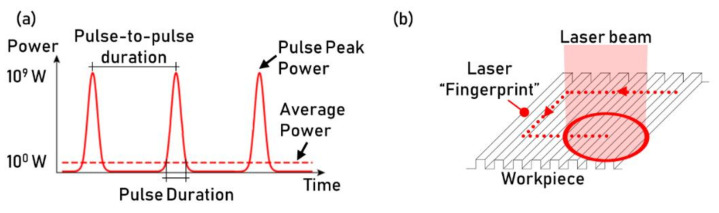
(**a**) Pulsing power typical shape and (**b**) the representation of the ultrafast texturing process.

Ultrafast texturing is a recent development in laser technologies, which has many peculiarities and allows machining of different textures. Ultrafast lasers are pulsed lasers characterized by a duration as small as units of femtoseconds and frequency ranging between one to several hundreds of kHz. Ultrafast laser systems are characterized by low power characteristics (i.e., units of Watts); however, the use of high power light pulses allows for the high values of energy being focused on the workpiece surface. In general, pulse powers exceed the gigawatts range (Figure 9a).

The extremely powerful pulses have a complex interaction with the workpiece surface that results in various types of textures [33]. The textures obtained using this laser texturing approach are commonly referred to as Laser-Induced Periodic Surface Structures (LIPSS) [33]. The high power ablates the material, which absorbs most of the energy delivered to the surface, limiting the HAZ dimension. Indeed, a thin layer (approximately 1 micron) of the bulk material is affected by the texturing process.

Ultrafast laser texturing exploits the complex interaction between the light pulse and the workpiece surface to machine textures with features that can be much smaller than the laser spot size and even smaller than the laser light wavelength [34]. The laser pulsing on the workpiece leaves a fingerprint of its nature in the form of a texture (Figure 9b). The generated textured is typically characterized by ripple-like structures, which geometry and regularity is typically characterized using a combination of Atomic Force Microscope (AFM) and Scanning Electron Microscope (SEM) [35]. The ablation mechanism involves the interference between the laser wavelength and the surface-associated wave. Ultrafast texturing is a complex phenomenon that occurs for a narrow range of laser fluence. The ablation threshold is specific for each material and should be controlled and identified for the designed optical setup [36]. The texturing process is affected by the characteristics of the beam and the process parameters. The textures’ main characteristics that can be controlled are features dimensions, shape, pitch distance, directionality, and regularity. The typical sizes of the structures range from several tens of nanometers to hundreds of microns. The structures’ size is strongly linked to the energy delivered to the surface (i.e., fluence) and the light wavelength. In particular, shorter wavelengths typically lead to smaller textures. For LIPSS structures, the pitch and directionality of the pattern are controlled by the polarization and the wavelength of the laser beam [37]. Good homogeneity of the texture can be obtained by optimizing the surface scanning parameters [38].

#### 4.1.4. Direct Laser Interface Patterning (DLIP)

**Figure 10:**
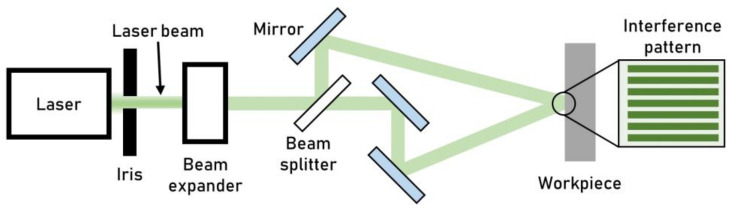
Schematic diagram of the DLIP process setup.

Direct Laser Interference Patterning (DLIP) exploits two or more laser beams to create an interference pattern on the workpiece surface, which ablates the workpiece surface, creating a texture. The development of small optical heads offers the possibility to process planar surfaces and complex three-dimensional products. The primary beam is split into two or more beams using beam splitters. Each generated beam is then sized and shaped to achieve the desired intensity (Figure 10). DLIP does not require focusing on the workpiece since the energy is sharpened in the desired areas by the interference field. The number of the interfering laser beams and their intercepting angle determine the shape and the spatial period of the intensity distribution pattern on the workpiece. The interference field results in high-temperature gradients, which ablate the material, producing a surface pattern.

DLIP can produce tiny textures, which can have the size of the laser beam wavelength. A large variety of pattern geometries can be engraved by tailoring the interference pattern as a function of the number of beams and their properties (e.g., polarization, intensity). With the use of ultrafast laser, hierarchical patterns can be machined with high control over the pitch. Hierarchical patterns are textures that present two or more patterns one over the other. The laser beam interference field machines the structures with the bigger pitch. Patterns with smaller pitch are obtained exploiting the phenomena discussed for the ultrafast laser patterning [39].

### 4.2. High-Energy Beam Machining

High-energy beam machining exploits different beam energies to melt and vaporize the workpiece with high accuracy and precision. The technologies described in this section are considered advanced manufacturing technologies and are implemented for high-end applications requiring high machining accuracy at the submicron scale. The MRR of these technologies is very low, and the equipment cost is high. However, high-energy beams can be used to texture hard materials at a submicron scale [25]. The techniques differ by the beam medium exploited: electrons or ions. Since the machining process needs to be in a high vacuum environment, the workpiece dimensions are typically limited to the vacuum chamber’s size.

#### 4.2.1. Electron Beam Machining

Electron Beam Machining (EBM) is a machining process that exploits high-velocity electrons to melt and vaporize the metallic workpiece surface. The electron gun utilizes high voltages from 50 to 200 kV to accelerate the particles to 50–80% of light’s speed. The process requires a high-vacuum environment to avoid any interaction with the electron beam. The electron beam is generated from the cathode and is energized by passing through the anode. The beam is then shaped and focused by the diaphragm, the magnetic lens, and the deflection coils. When the beam hits the workpiece, high temperatures are reached on the surface, and the material melts and vaporizes. The high vacuum pump system continuously evacuates the generated debris. The electron beam can be accurately focused onto the workpiece surface, allowing to machine feature down to tens of nanometers. The MRR of this process is very low, up to 1 mm^3^/min [19].

#### 4.2.2. Focused Ion-Beam Machining

The Focused Ion-Beam Machining (FIBM) process exploits ions as the beam medium. The process functions are similar to the EBM process. The ions are typically extracted from a gallium (Ga) source using extraction electrodes [40]. The condenser lenses and the objective lens shape and focus the beam on the workpiece surface. The selection of the beam diameter and the probe current is mechanically ensured by the ion current selection apertures and measured by the Faraday cup. The beam astigmatism is corrected using the stigmation octupoles. Ultimately, the objective lenses focus the ions on a small and precise spot on the surface. The beam current profile on the workpiece determines the machining accuracy and precision [41]—generally, the smaller the beam diameter, the tighter the resolution. However, the beam spot size is limited by the chromatic aberration resulting from the ion source’s energy spread. The ion beam can be focused on tiny spots, down to 10 nm. The MRR can be enhanced by tilting the ion beam to the surface. The effect of changing the incidence angle is a higher momentum transfer between the ions and the workpiece surface atoms, resulting in more ejected particles. As anticipated, the MRR is low—down to 0.1 mm^3^/min—and can be more economical for hardened steels [42].

## 5. Additive Manufacturing

### 5.1. Lithography

**Figure 11:**
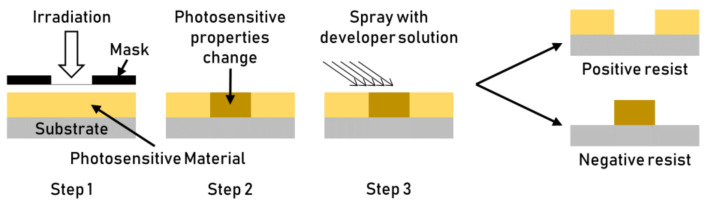
Working principle for lithography techniques.

Lithography-based processes exploit masks to create a texture on a photosensitive material by selective irradiation. The resist material (often an organic polymer) is deposited over a substrate (usually silicon) and then irradiated (step 1, Figure 11) using a mask with the desired pattern. The irradiated photosensitive material changes its properties (Step 2, Figure 11). Depending on the deposited material’s chemical nature, it can either be destroyed or hardened by irradiation. The irradiation causes crosslinking for negative resists and bond breaking for positive resists. The unexposed (for negative resists) or the exposed (for positive ones) areas are removed with this mechanism. The photoresist that is not cured is removed using a solvent (Step 3, Figure 11). A developer and a suitable solvent are finally utilized to develop the resist pattern [43]. Depending on the pattern’s required resolution, different lithography processes can be completed using varying irradiation sources (UV light, X-rays, ions, or electrons). The resist pattern is then used as a mask. The etching of the substrate’s exposed areas, or through metal deposition of a new layer, creates a textured surface. The lithography process was initially developed for the electronics industry. However, it has recently been adopted for more applications, such as the texturing of plastics manufacturing tools.

The lithography process’s resolution is mainly affected by the incident irradiation and can vary from 10^0^ to 10^2^ nanometers. For example, for optical lithography (photolithography) applications, the resolution is linked to the light wavelength and is limited by the diffraction limit of the light (i.e., about half of the wavelength). For this reason, light sources with shorter wavelengths, from UV light to X-rays, were used to improve accuracy. The resolution of UV lithography (i.e., several hundreds of nanometers) can be enhanced to nearly a hundred of nanometers using Deep Ultraviolet Lithography (DUV lithography), or to a few tens of nanometers with X-ray lithography [44]. However, expensive optics and resist materials are required when using shorter wavelengths, leading to higher processing costs. Despite the high investment costs for a photolithography facility (reported to be around $30 MLN), it is a well-established technique for large-scale production due to its high throughput. New optical systems are being developed to achieve sub-wavelength patterning resolution.

Other techniques were developed to overcome the resolution limitations of optical lithography using electron irradiation (Electron Beam Lithography, EBL), or ion irradiation (Focused Ion Beam Lithography, FIB). The resolution of these technologies can be as low as 101 nanometers; however, the throughput is limited. Compared to UV lithography, the pattern can be directly machined on the surface workpiece without a mask. The low performance of EBL and FIB limits their application for large-scale production. However, they are still used for the nanofabrication of plastic injection molding tools, which might require tiny features [45].

### 5.2. LIGA

**Figure 12:**
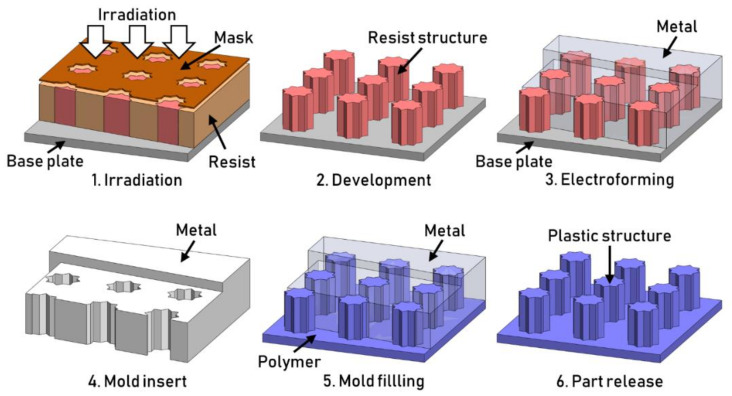
LIGA technique working principle and manufacturing steps.

The LIGA process (German acronym for Lithographie, Galvanoformung, Abformung, which can be translated as Lithography, Electroplating, and Molding) is a process used for the manufacturing of textured substrates. The technique allows the fabrication of high-aspect-ratio (up to 100:1) nano-features. The process was developed at the now-called IMT (Institute for Microstructure Technology) in the late 1980s. The process consists of several steps (Figure 12), namely (1) surface irradiation, (2) the development of a pattern through X-ray lithography, (3) electroplating the pattern to obtain a metal (for example, nickel) nano-structured mold tool, and (4) molding through the embossing or injection molding of a thermoplastic resin (5) to mass-manufacture textured parts (6). The dimensional resolution of the process is mainly linked to the X-ray lithography process. High values of the aspect ratio for the structures are achieved by introducing a working mask [46]. Fabricating the working mask is more expensive because it introduces several more steps to the process. The working mask is made by lithography and gold electroplating. The high thickness (e.g., 25 µm) of the electroplated gold layer enhances the X-ray absorption of the mask, allowing the development of high aspect ratio microstructures (HARMs). The textures generated using LIGA are characterized by high parallelism of the side walls, which, combined with the aspect ratio, can lead to ejection problems when molding [4]. However, it is possible to add draft angles to the structures’ sidewalls using oblique X-ray exposures of the photoresists. Draft angles of 3° were successfully manufactured [47]. It should be mentioned that this technique has the undesirable effect of reducing the nickel’s strength and hardness by a factor of approximately 2. Alloys with higher temperature properties are introduced when using this technique to substitute nickel [47].

### 5.3. Maskless Additive Manufacturing

**Figure 13:**
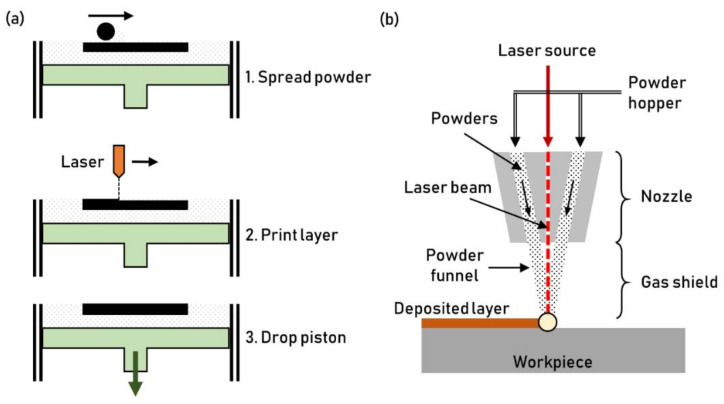
Working principles for (**a**) Selective Laser Melting (SLM) and (**b**) Direct Energy Deposition (DED).

Additive Manufacturing (AM) technologies are used to texture plastic manufacturing tools. AM of metals exploits the thermal energy, delivered by a laser beam, to locally melt a metallic powder. The two leading AM technologies for manufacturing textured surfaces are Selective Laser Melting (SLM) and Direct Energy Deposition (DED). Both processes melt the powder onto a substrate to form the AM part layer by layer. The two methods differ due to the powder depositing mechanism. SLM is a sequenced process: the powder is firstly spread over the surface (Step 1, Figure 13a); secondly, the layer is printed by scanning the newly formed powder surface (Step 2, Figure 13a), then the substrate steps down to allow the subsequent powder layer deposition (Step 3, Figure 13a). At the end of the process, the fresh powder is removed [48]. Conversely, DED exploits a constant powder funnel sprayed on the workpiece surface (Figure 13b). During printing, the laser selectively melts the powder provided by the nozzle.

The most commonly used material for these AM technologies is stainless steel (AISI 316 L) [49]. The powder particle size is typically in the micrometers range. In general, the smaller the particle is, the finer the surface finish will be. However, the use of fine powder leads to worse melting behavior, resulting in structural defects, such as pores and voids.

The microstructures achieved by powder melting typically show low tribological and mechanical properties due to defects and voids. A thermo-chemical method can be used to enhance the structural properties of the texture. By plasma nitriding of 316L stainless steel S-phase and CrN, a compound layer can be formed on the surface. Plasma nitriding does not only change the surface chemistry of the material, but it also creates a hard ceramic-based nitride layer on the surface, called the compound layer. While the steel lattice is modified, surface textures geometries are minimally affected by the treatment. However, treated textures show higher surface roughness. The compound layer of nitrided samples can show high shear resistance, sometimes doubling the hardness with respect to the untreated textured tool [50].

Micrometer and sub-micrometer textures with unique geometries can be obtained using AM technologies. SLM can achieve finer texture geometries, down to about 100 μm [49]. As the pattern dimension reduces, finer particles have to be used, and the powder evacuation at the end of the process may become complicated. DED can be used to create texture on complex geometries, by direct deposition and sintering of the particles. A careful process parameter optimization must be conducted to improve the structure accuracy and mechanical resistance while containing the printing time. Laser power, powder flow rate, and printing patterns are the key parameters that need optimization. By reading the current melt temperature, laser heads equipped with adaptive process control (APC) can actively tailor the laser power to uniformly build the structures. The APC consists of a CCD camera to monitor the melt temperature for a close-loop feedback system on the laser power [51].

### 5.4. 3D Printed Soft Tooling

The commercial metal printing processes presented above are characterized by resolution limitations for texturing applications. When considering polymer-based 3D printing technologies, they can be split into two groups: (1) print methods which share resolution limits with metal counterparts and (2) processes with acceptable resolution, but insufficient material properties [52]. Selective laser sintering (SLS) and multi-jet fusion (MJF) are examples of polymer-based additive manufacturing technologies characterized by resolution limitations similar to Electron Beam Melting and Direct Metal Laser Sintering. Indeed, surface roughness can be challenging as typical particle sizes range from 15–150 µm [53]. Resolution challenges also limit the utilization of fused deposition modeling (FDM) for texturing. In fact, the print stratification effects and minimum resolution are both a function of the filament diameter deposited by the printer, often limited to a minimum resolution of 100 µm [54]. Moreover, the filament deposition leaves voids between passes, which yields compression strength below the requirements for injection mold tooling.

For systems which do exhibit acceptable process resolution, resin properties are often the challenge when considering molding applications. VAT photopolymerization processes show promise in terms of resolution with laser-based Stereolitography (SLA) systems and Digital Light Processing (DLP)-based SLA systems reporting minimum features of 76 [55] and 35 µm [56], respectively. For SLA processes, engineering resins have been developed, and some materials have heat deflection temperature (HDT) and flexural modulus properties suitable for molding applications. Zhang et al. demonstrated the production of a mold insert with micro features using a high-temperature resin in a DLP process, achieving successful replication for about 80 cycles [57].

## 6. Advantages and Limitations

Many techniques have been developed to address the texturing needs of a large variety of stakeholders. Mechanical machining, electro-chemical etching, thermo-electric, and additive processes have been applied in different fields due to their unique advantages. The selection of the most appropriate texturing process depends on the application and requires combining different engineering skills in materials, manufacturing processes, product design, quality control, and more. The texturing of a plastic product affects the customer perception, and thus changes its market value. The successful manufacturing of a textured product requires a deep understanding of the different texturing technologies. In this section, the comparison of the advantages and disadvantages is presented to allow engineers and designers to select the most appropriate and effective technology.

### 6.1. Geometric Texture Parameters

The most critical parameter to consider when selecting a texturing technology is the dimensions of the surface features. Different scale features within the texture result in a wide range of diverse functionalities. Depending on the desired product properties, texture dimensions can range from millimeters (10^−3^ m) to nanometers (10^−9^ m). The successful manufacturing of textures at different scales requires considering different technologies. In fact, not all texturing technologies can be used to manufacture a specific scale texture. However, each technology is characterized by its dimensional range.

Textures are also categorized as random and patterns. The former refers to surface features that are not regularly disposed over the product surface. The latter defines textures that have specific features orientation, location, and reciprocal distance. The ability to generate an accurate pattern rather than random structures is a specific characteristic of the different texturing technologies. The characterization methods for the two types are also different. The topography of random textures is well characterized using surface roughness values. Instead, regular patterns require more accurate and broad characterization, which might require the quality analysis of individual features. In general, patterns tend to be bigger than random textures because of the need for regularity and the more stringent quality criteria.

Different geometrical parameters can be defined to describe a texture [1]. The most important are:

- The spatial pitch of the pattern, i.e., the distance between consecutive features.
- The features’ cross-section geometry, such as their diameter or width.
- The feature height or depth, i.e., the distance from the substrate to the top or bottom of the features.
- The aspect ratio, i.e., the ratio between the feature height or depth and its width.

Among all dimensional parameters, the aspect ratio of the texture is the most significant when considering the manufacturing of a textured plastic product. For most applications, aspect ratio correlates with surface functionality, thus being crucial for selecting and comparing texturing technologies. The aspect ratio is one of the most critical texturing characteristics and constitutes a manufacturing constraint in applying the specific texturing technology to a broader range of applications [1].

**Figure 14:**
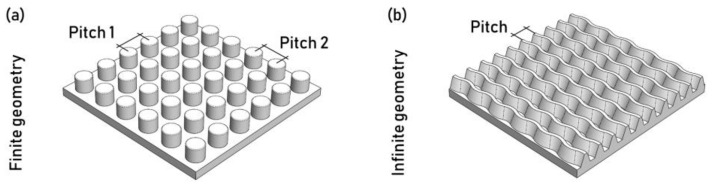
Schematic of (**a**) finite geometries pattern and (**b**) infinite geometries pattern.

The feature geometries can be divided into two main categories: finite and infinite geometries. Finite geometries are defined by distinct features that all have dimensions in the same order of magnitude. Examples are distinct pillars, holes, bumps, and more (Figure 14a). Infinite geometries are texture features characterized by a dimension being substantially larger than the others (Figure 14b).

**Figure 15:**
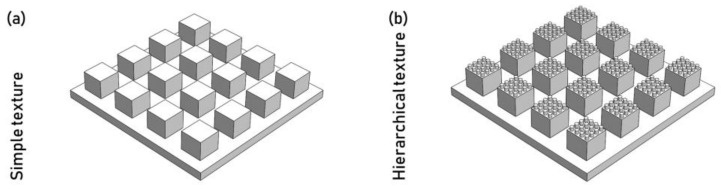
Schematic of (**a**) a texture with structures of the same order of magnitude and (**b**) of a hierarchical texture.

Some texturing technologies can allow the manufacturing of hierarchical surface features. Hierarchical textures are characterized by overlapping two textures, one on the other (Figure 15) [58]. This type of texture allows different product functionalities due to the presence of features at different scales. Typically, the larger and smaller features have dimensions in different orders of magnitude.

The functionality of a textured surface is inherently linked to its quality. Quality criteria for textures include features, shape, dimensional, and positioning accuracy. Shape accuracy is typically limited by the presence of burrs, undercuts, and recasts. Even if such defects do not compromise the texture, they increase the “noise” on the surface. The “noise” is usually only qualitatively addressed, since the standard surface roughness parameters (e.g., Ra or Sa) are not affected by the noise level.

### 6.2. Process Texturing Capabilities

The ideal texturing technology should accurately manufacture the desired surface features onto a large 3D free-form surface, for any material, in a reduced number of steps and at a low cost. Such texturing technology does not exist, and the appropriate technology must be identified among the many available. However, the definition of the ideal texturing method allows the outlining of a texturing process’s capabilities. In general, four main classes of requirements can be identified:

1. Texture scale—the overall dimension of the desired features has to be defined in agreement with the different texturing technologies’ capabilities and limitations;
2. Geometrical flexibility—the texturing technology property of generating features on surfaces with different complexity, from planar to three-dimensional to free-form needs to be considered;
3. Process–property relation—the ability to create specific texture features on the selected material needs to be evaluated. Then, the damage produced during processing should be considered;
4. Economics—different texturing technologies are characterized by significantly different costs but they also offer different possibilities.

### 6.3. Achievable Shapes and Dimensions

The main parameter that has to be considered when selecting the texturing technology for a specific application is the texture dimensions. Most texturing technologies are capable of generating surface features within a particular dimensional range. For example, aesthetic functionalities are typically realized with millimeter range features (e.g., using chemical-etching), while functional textures are in the micrometer range (e.g., using LIGA). Thus, the application would require selecting different texturing technologies and consideration of different processing-related issues. The texturing technology is also defined as the shape of the designed features, controlled mainly by the presence or absence of a mask in the process. Through-mask processes are used to accurately control the features’ cross-section, while the height/depth is controlled by removing the unmasked material. This approach results in vertical sidewalls, which have no draft angles. For this reason, these structures are commonly referred to as 2.5-dimensional. The main characteristic of this type of structure is its aspect ratio [59]. Conversely, maskless processes allow the generation of fully three-dimensional textures.

- Micro-milling technologies are used to machine three-dimensional textures with features that may be as small as 10 microns with aspect ratio of 3:4 [10]. The CNC control guarantees high shape consistency (i.e., the capability to produce the same shape) across the texture. However, the shape accuracy (i.e., the capability of producing the desired shape) is affected by the material grain size and the presence of burrs. The latter is very difficult to remove at the micro-scale. The machining of smaller grain size steel showed much higher shape accuracies [60]. Process modifications, such as diamond tool micro-milling or ultrasonic-assisted machining, were introduced to limit burrs formation.
- In the AJM process, the mask defines the minimum feature dimension, typically in the order of 100 μm. The mask material has to be tough enough to withstand the erosion of the abrasive jet. Aspect ratios for this process are usually low (around 0.5). The shape accuracy can be enhanced by choosing different abrasive particles. The size, the shape, and the material hardness of the abrasive have a substantial effect on the shape accuracy [21]. Smaller particles with few sharp edges and relatively low hardness can produce better textures.
- A large variety of structure shapes can be obtained using µEDM with dimensions down to tenths of microns. The aspect ratio is mainly constrained by the flushing efficiency [26] and the maximum values are about 2. Indeed, flushing is increasingly challenging for a higher aspect ratio, and the adequate electrical conditions for the spark may not be reached. Flushing can also affect the stability of slender electrodes when the flushing velocity is increased. The feature shape accuracy is mainly linked to the spark crater and tool wear, which can be minimized by reducing the spark energy. Tool wear is avoided in ECMM, resulting in higher texture consistency. The machinable features and aspect ratios are comparable to µEDM for the conventional process, and they depend on process parameters like voltage and electrolyte purity. Ultrasound-assisted ECMM was introduced to push the feature dimensions down to 1 µm and the aspect ratio to 3 [29]. The tool vibration enhances the electrolyte flushing and heat removal.
- Masked processes, such as TMECM or chemical etching, are used to manufacture 2.5-dimensional textures. The achievable aspect ratio is about 1, but textures with lower aspect ratios are usually machined with these processes. The electrolyte or the etchant is flushed over the surface to maintain a homogenous material ablation and obtain better structure consistency throughout the entire surface. In both processes, etching can create undesired undercuts by removing material under the mask. Hence, larger masks are used to consider this effect.
- Laser-based technologies generate different features based on how the beam light is delivered and focused on the surface. In laser writing, a large variety of features as small as 20 µm can be obtained, with an aspect ratio up to 3 [31]. The interference phenomena that characterize ultrafast laser texturing and DLIP result in smaller feature dimensions, down to 100 nanometers. For these technologies, the texture dimensions depend on the light properties (i.e., wavelength) and process conditions (i.e., irradiance and scanning speed). The upper limit on feature size is around 100 µm for LIPSS obtained using ultrafast laser texturing and 500 µm for DLIP [39]. The shapes obtained with these technologies are limited due to the tight correlation with laser light properties, such as polarization and wavelength. Ultrafast laser texturing and DLIP can be used to generate hierarchical textures. The primary shape defects of laser-based texturing technologies are recasts around the engraved area and thermal micro-cracks. As a consequence, texture consistency and shape accuracy are the main challenges.
- When a mask is used for laser texturing, the minimum feature dimension is constrained by the diffraction effects when the laser light crosses the mask. Features dimensions as small as 5 µm can be engraved with aspect ratios smaller than 0.5. The texture consistency along the surface is typically high but the shape accuracy is limited by recast and thermal micro-cracks. High-energy beam techniques are used to generate three-dimensional structures with small dimensions (i.e., down to 100 nm for FIBM) with aspect ratio not bigger than 1. The machined features are typically accurate and precise.
- A wide range of three-dimensional surface features with dimensions as small as 100 µm can be manufactured using additive manufacturing technologies. DED’s working principle makes the achievable features bigger and less accurate than those obtained using SLM. SLM offers the possibility of achieving higher aspect ratios (i.e., up to 4 for SLM and up to 2 for DED). The minimum feature dimensions and the shape accuracy are affected by the powder particle dimensions, which are typically not smaller than a few tens of microns [49]. Typical defects are the bridging between different surface features, balling, non-evacuated powders, or internal voids.
- Lithographic techniques manufacture 2.5-dimensional features with high aspect ratios, with dimensions as small as few nanometres. Simple three-dimensional features can be manufactured using the LIGA technique. However, features cannot be smaller than 500 nm. These techniques result in textures characterized by high consistency and accuracy.

### 6.4. Geometrical Flexibility

The texturing of plastic products and manufacturing tools often requires the generation of surface features on three-dimensional geometries. Textures have been successfully applied to surfaces of various shapes, from simple flat to hard-to-reach surfaces. The selection of the most appropriate texturing technology requires understanding the geometrical flexibility of the different working principles. The most crucial factor to consider is the need for a mask; mask-assisted processes are more challenging to implement for complex shapes.

- In mechanical machining technologies, tool movements are CNC controlled along multiple axes. Thus, the equipment is highly flexible and can be used to texture complex surfaces. However, milling-based technologies are limited by the length-to-diameter ratio of the cutting tool [10,15]. Abrasive jet machining is more flexible when texturing hard-to-approach areas, as the jet can be optimized to travel a longer distance on the workpiece [21]. However, the generation of regular patterns requires a mask, limiting the ability to work on complex free-form surfaces. In comparison, abrasive jet machining can be used to obtain random textures on complex products.
- The use of a mask introduces geometrical limitations for electrochemical etching processes, such as chemical etching and through mask electro-chemical machining. Maskless technologies can be used for more complex geometries, such as micro electro-discharge machining and electrochemical micromachining. These processes can exploit simple shape tools or pre-patterned ones to texture different workpiece geometries [8]. In this case, the effective flush of the debris from the working area is crucial, which defines the limits for geometrical complexity.
- Laser-based thermo-electric engraving technologies allow good geometrical flexibility. Laser writing is the most flexible technology among the four laser-related technologies considered since the optical head is mounted on a five-axis CNC machine. When using an ultrafast pulsed laser, issues related to beam handling reduce the flexibility for ULT and DLIP. These technologies cannot exploit CNC machines because of the high pulse power. Hence, they use mirror systems to redirect the laser beam to specific surface locations. However, the need to deliver an unchanged laser beam to the workpiece surface makes this approach complex and limited. Indeed, ultrafast pulsed laser technologies exploit the laser beam’s light wave characteristics not just thermal energy. Similarly, high-energy beam machining techniques (i.e., EBM and FIBM) require advanced equipment to handle the beam, limiting the workpiece dimension and allowing the texturing of 2D surfaces only.
- Lithography and LIGA technologies allow texturing of planar surfaces only. Additive manufacturing texturing technologies have higher flexibility. For example, DED nozzles are often mounted on multi-axis CNC machines. However, substantial limitations should be considered for concave geometries’ texturing, due to the dimensions of the nozzle and the short powder jet length. The SLM process allows texturing free-form surfaces only when the product and the texture are realized in the same process. However, the processing times can be very long. Conversely, if SLM is used to texture an existing product, only planar surfaces are allowed but the texturing time is significantly shorter.

### 6.5. Material–Process Relationship

The generation of surface features on the workpiece can lead to material modifications at the surface. The type and entity of these changes depend on the relationship between the selected material and the texturing technology being used. Selecting the most appropriate texturing technology requires understanding the thermal and chemical modifications at the interface. The effects of texturing on the material properties are discussed considering the main types of defects, namely mechanical stresses, chemical modifications, the heat-affected zone, oxidation, porosities, and inclusions.

- Textures obtained with mechanical machining processes are characterized by residual stresses, affecting the texture strength and wear resistance. The tool contact creates these defects as a result of the ablation and material removal. Textures can be micro-milled on a wide range of materials, and the quality is mainly affected by the grain size. The edge radius of cutting tools can be as small as the grain size, and machining instabilities can occur. Hence, for high-accuracy applications, materials with finer grain size are preferred. Similar phenomena are also crucial in abrasive jet machining, in which the particle size, shape, and hardness, are selected to avoid the inclusion of particles inside the workpiece.
- Electro-chemical etching techniques result in stress-free textures. However, selecting the appropriate etchant for a specific substrate material is crucial to avoid chemical damage to the workpiece surface. The chemical modifications of the exposed surface are evident even after rinsing, but they are a concern only for a specific application (e.g., clean room tools). The chemical modifications of the surface are minor with ECMM. However, if inclusions characterize the metal substrate, those can affect the resulting properties.
- In the EDM process, the texture is generated by thermal phenomena, such as localized melting and vaporization. Thermal stresses on a superficial layer characterize textures obtained with this technology. However, structure and crystallinity can be modified further down in the workpiece, thus defining the Heat-Affected Zone (HAZ). Thermo-electric processes are always characterized by the presence of a HAZ on the textured surface.
- Laser-based texturing technologies typically show thermal micro-cracks and recasts. The fast cooling of the melted material over the surface that is not ablated produces micro-cracks. The condensation of vaporized material over the surface produces the recasts. Pulsed lasers were introduced to reduce the HAZ and enhance machining accuracy. Ultrafast pulsed laser processing is defined as a “cold process” because the HAZ can be neglected. However, surface oxidation can be a problem with these technologies. The HAZ is also minimal for high-energy beam machining, but EBM and FIBM are executed in a vacuum environment to avoid oxidation.
- Additive manufacturing texturing can only be carried out with materials that are not prone to oxidation, such as stainless steel. The oxidizing of the powders ultimately compromises the texturing process. Residual thermal stresses, micro-cracks, and internal porosities are the most common defects of this process. Porosities, inclusions, and residual stresses are common in the LIGA process because of the electroplating with nickel.

### 6.6. Economics

The product market value and the texturing methodology determine the economics of texturing applications. The impact of texturing on a commercial product value is not related to the engineering manufacturing aspects. However, the texturing costs can be associated with the following:

1. Cost of the materials needed to generate the texture (e.g., metal powder for an SLS process or nickel for LIGA);
2. Cost of the equipment and auxiliary needed for a specific texturing technology;
3. Cost associated with the texture generation (e.g., required to operate an EDM machine or consumable used in a chemical-etching process);
4. Quality control and characterization costs (e.g., access to advanced microscopy equipment to characterize a directly laser-written texture).

The different texturing technologies have different characteristics, which need to be analyzed separately. The desired texture eventually determines the comparison of the economics of different products. However, some major economical parameters for each technological group are described below:

- Mechanical machining texturing uses specific multi-axis CNC machines with high-spindle speeds, a rigid structure, and a temperature-controlled environment. Machining tools typically have a short life due to high machining speeds and small tool dimensions. Harder tools (e.g., coated or diamond tools) are often exploited to decrease the tool wear despite the higher cost. Moreover, micro-milling tools are significantly more expensive (i.e., up to 10 times higher) than conventional ones. The machining process is characterized by low MMR and so low texturing speed. The latter depends on the material hardness, texture complexity, and workpiece geometry. Overall, the costs are mitigated by the short setup time and the near-net-shape that are typically manufactured. These processes are characterized by high energy efficiency and a low environmental footprint. The primary ecologic concern is the lubricant, typically filtered and recirculated by the machine, but it must be correctly disposed of at the end of life.
- Multiple steps and low MMR characterize electrochemical etching. However, the economics are mitigated by the ability to create textures on large areas. The need for chemical solvents makes the operation costs higher, as specific health and safety procedures need to be implemented. In ECMM, multiple-axis CNC machines are used, resulting in higher MMR. Moreover, the lack of contact between the tool and the workpiece reduces the tooling wear and costs. During processing, handling, and disposal of electrolytes has to be considered. However, it is less hazardous for operators and the environment compared to chemical etchants. µEDM is a net-shape texturing technology, but the manufacturing of small and low surface finish textures requires small spark energy that leads to low MMR. Increased throughput is achieved with multiple roughing passes before finishing. With this strategy, the depth of the craters obtained with roughing might limit the achievable surface finish.
- Laser-based texturing is controlled using multiple-axis CNC machines. The equipment cost is high for ultrafast lasers, especially for femtosecond laser sources which require moving lenses to create complex optical paths. Texturing speed varies significantly with specific laser technologies. Masked-laser texturing has higher throughput than laser writing due to its ability to manufacture larger areas. Ultrafast laser texturing and DLIP exhibit excellent texturing speed, considering the achievable texture dimensions (from 10^−2^ to 10^2^ μm). Laser texturing is an environmentally sound technology that does not require handling any hazardous substance.
- EBM and FIBM require advanced and costly equipment. High-vacuum chambers are used to focus high-energy beams on the surface. The expensive setup is also limited when considering the size of the workpiece. Moreover, texturing speed is extremely low, and energy efficiency is poor. The economics of this technology are justified for high-end products that require unique three-dimensional structures with high accuracy.
- Lithography techniques are costly because of the multiple steps required for texturing. Moreover, the equipment and consumables (such as masks) are expensive and handled by specialty workers. The technology owes its large diffusion to the ease of scale-up and high throughput. Manufacturing facilities for photolithography require high investments (about USD 30 million) but allow large-scale manufacturing.
- Powder-based additive manufacturing processes are characterized by high setup, processing, and material costs. A protective atmosphere must be created within a CNC machine to avoid oxidation of the powder. Powder handling involves health safety concerns, thus requiring specific handling tools and storage. In texturing tends that are slow, however, the design flexibility makes the technology attractive for a few specific applications.

## 7. Applications of Texturing in Injection Molding

**Figure 16:**
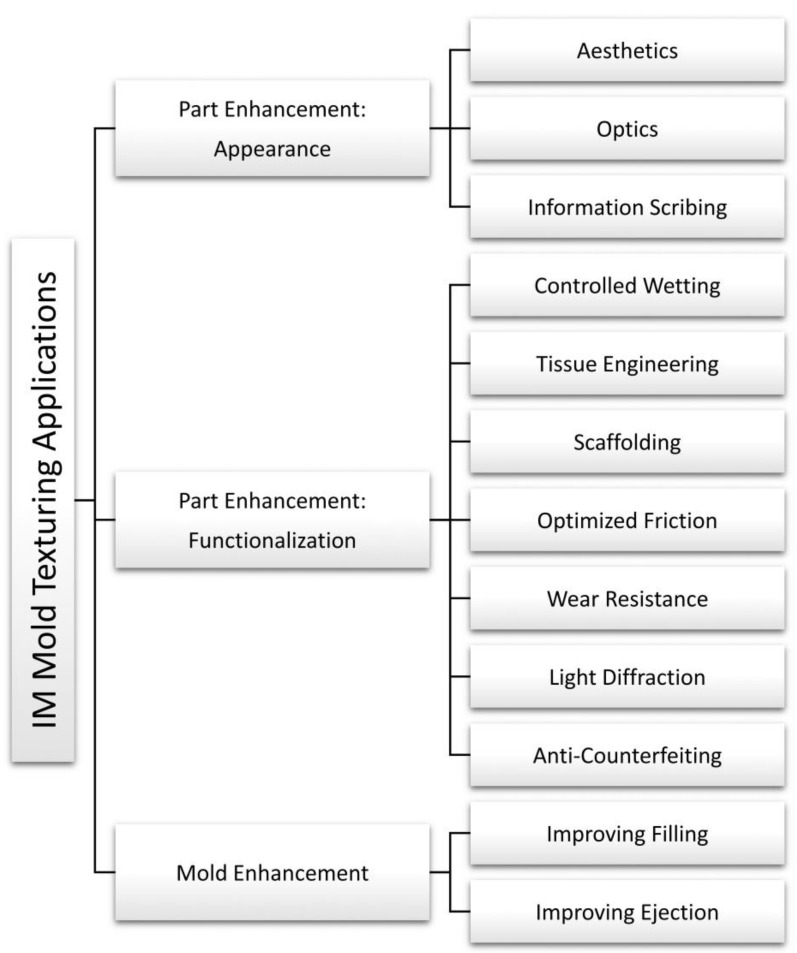
Applications of surface texturing in injection molding and their functionalities.

Texturing finds different applications in injection molding technologies, ranging from plastic products to mold functionalization. Textures have been used to improve the appearance and functionality of plastic products, allowing significant opportunities for plastic manufacturers. More recently, the increased availability of texturing technologies has opened up opportunities for mold surface functionalization. The generation of micro- and nano-scale features on tool surfaces has improved the filling and ejection in plastic injection molding. Figure 16 summarizes the field of application and the functionalities of texturing for injection molding. The texturing technologies presented in this work have been used for molding applications with different levels of success. While some are well-established (e.g., chemical etching or micro EDM), others require more application-specific development in conjunction with polymer processing considerations.

### 7.1. Aesthetic Texturing

A plastic product’s aesthetic appearance can be modified by adding textures in the sub-millimeter on a micro-scale range. Smaller features, not visible to the human eye, are typically used for other applications. The texture design process is often based on creativity and marketing. However, the aesthetic surfaces often mimic natural textures like leather, wood, or animal skins. Other surfaces are textured to create grained, dull, soft, smooth, structured, or rough-shaded effects that are perceived as elegant cosmetic surface finishes. These textures change the customer’s perception of a product, thus creating market value. Examples range from the interior surfaces of different vehicles to PET soda bottles. However, these surfaces’ applications are limitless and have been successfully implemented in automotive, electronics, packaging, and more industries [61].

The features’ dimensions and design determine the selection of the most appropriate texturing technology for aesthetic applications. The most commonly used technology for aesthetic texturing is chemical etching due to its ability to work with large surfaces. However, more recently, laser-based technology has also been implemented thanks to its flexibility and smaller environmental footprint. For example, Brenner et al. used laser to texture a large and 3D mold used for an automotive application [62].

The successful replication of a textured injection mold requires particular processing consideration. Specifically, high mold temperatures may be required to replicate the texture geometry completely. These applications are supported by rapid heat cycle molding technologies, which allow heating of the mold above the polymer transition temperature during filling and rapid cooling before ejection [2,63].

### 7.2. Optics Functionalization

Surface texturing finds application in plastic products that require specific optical properties. Optics applications require replicating mold textures using transparent polymers, allowing the manufacturing of different optical components. Examples of micro- and nano-textured optical components include lenses, diffractive optical elements, diffraction gratings, blazed gratings, slanted gratings, diffractive diffusers, optical diffusers, and beam splitters. The industries implementing textured plastic lenses in their products are mainly automotive and electronics. In both industries, semiconductors for illumination and sensing systems led to the development of micro-lenses used as diffractive elements. The light beam generated by LED (Light Emitting Diode) or OLED (Organic Light Emitting Diode) is shaped by surface features when passing through the lens. The most common products are headlights for vehicles or camera lenses for smartphones. More advanced applications include components for photovoltaic systems, as manufactured by Fritz et al. using lithography for the insert texturing and hot embossing for replication [64].

The most common type of texture used for this application is micro-scale prisms (i.e., depth and width in the order of 10^2^ μm and radius of 10^1^ μm). The surface features must have a high surface finish and accurate dimensional tolerances. Hence, micro-milling, UV lithography, and laser writing are the most commonly used texturing technologies. Other technologies, such as μEDM, are more challenging because of their inherently high surface roughness [65]. The master tools’ surface structures are generally coated to increase their wear resistance and promote the release of the polymer at the end of the replication (Saha et al., 2016). The textured tools are replicated on a large scale using different manufacturing processes, such as injection molding, hot embossing, and roll-to-roll.

### 7.3. Information Scribing

Information scribing is an inexpensive, durable, and secure approach adopted to trace a product. Branding with appealing logos or artistic textures is widely used for both consumer and high-end products. By engraving a brand name, serial number, or other legal marks on a product, a company not only reinforces its brand image but also protects its name. Counterfeiting can be a challenge to a company’s ability to protect its name and brand identity. An engraved serial number or a complex geometry on a mold ensures a level of security for the company [66]. Moreover, engravings report information regarding the polymer grade, recyclability, and end-of-life strategies. The texturing technologies adopted for information scribing are mainly laser-based due to their ease of use and flexibility [67]. Information scribing range from letters or logos engraved using laser ablation to nano-scale structures used for surface coloring to micro-scale counterfeiting features.

### 7.4. Structural Colors

Texturing is an effective solution to create light diffraction effects on the surface of plastic products. Submicron scale patterns diffract the light when their pitch dimension is close to the visible light wavelength (i.e., 380–740 nm). Depending on the size and orientation of the structures and the angle of orientation of the incident light, different shiny shades of colors are visible throughout the entire visible spectrum [33,68]. The diffraction effect is similar to the one that can be seen on CDs and DVDs, but differently, as only one color is shown at a specific angle. Homogenous textures with long ripples are required to show structural colors, and smooth areas may alternate them to increase the shiny effect [66]. The diffraction effect is mainly obtained with laser-based texturing technologies, such as ultrafast femtosecond laser ablation.

### 7.5. Controlled Wetting

Textures with micro- and nano-scale features are used to modify and control a surface’s wetting properties. The wetting properties of a surface define its interaction with a liquid, and the contact angle is the most representative indication of the wetting behavior. The most famous example of the effect of texturing on surface wettability is found in nature on lotus leaves, which easily repel water. The presence of micro- and nano-scale hierarchical features modifies the interaction of the surface with liquids.

Adding a texture to a product can lead to hydrophobic behavior [69]. Engineering applications that exploit this phenomenon include hydrophobic [70], hydrophilic, icephobic [71], anti-fogging, self-cleaning [72,73], anti-fouling [74], and more functional surfaces [75]. Oleophobicity (i.e., the ability of a surface to repel oils) and amphiphobicity (i.e., the surface’s ability to repel both water and oil) can also be controlled and enhanced using texturing.

Hierarchical textures are commonly exploited to control wetting properties [76]. Two substantially different scale patterns define the two overlapped textures. The larger one is typically characterized by features of the order of 10^1^ μm. The smaller textures, realized on top of the other, are typically smaller than 10^2^ nanometers. For example, Wang et al. used a picosecond laser to generate different textures on mold steel with super-hydrophobic properties [34]. Similar surfaces were replicated using injection molding from inserts textured using EDM [77]. It should be noted that the polymer–texture interaction is also significant in these applications because the effect of patterning the surface enhances the polymer’s intrinsic surface properties. Typically, polymers show a low wetting with water that can be further improved, obtaining a more hydrophobic behavior (i.e., reducing its surface energy) [59]. Moreover, the polymer’s rheological and thermal properties influence the ability to achieve complete replication using a specific polymer processing technology [63].

Hierarchical textures have been manufactured using different texturing technologies. The most critical aspects of their manufacturing are related to the submicron scale texture generation, which needs to fit on the first texture accurately. The use of laser-based technologies allows excellent flexibility and control over small patterns [78]. High-energy texturing technologies have also been used, but they are typically carried out in clean-room environments.

### 7.6. Biomedical Functionalization

Textured surfaces find several applications in biomedical devices manufactured with polymers. Surface functionalities have been introduced to modify interactions with biological substances, such as cells, tissues, fluids, and more. The topography modification can significantly impact the biological response of the surface. Textured surfaces have been used to improve the interrelation between implants (e.g., pins, screws, rods, clips, etc.) and the surrounding tissue [79,80]. Other examples include tissue [81] and cell engineering scaffolds [82], microfluidic devices [83], organ-on-chip [84], and surgical tools. Texturing technologies are also being used to enable the large-scale manufacturing of microneedles [85,86].

The application of micro- and nano-structured plastic products for medical devices are expected to continuously grow, owing it to the wide range of properties that can be obtained using polymers. Biomedical applications exploit very different texturing technologies, and the most appropriate is typically selected based on the specific product.

### 7.7. Controlled Friction

The tribological properties of polymers are often poor, thus limiting their use for dry contact applications. In the industry, the problem is solved using lubricants (solid or liquid) or by changing the resin formulation. Surface texturing is an alternative to these techniques. The surface features reduce the contact area, entrap the wear debris, and improve surface/lubricant contact [87]. Depending on the lubrication condition and texture geometry, the tribological effect of a textured surface varies significantly. Thus, it is essential to design the right texture for a particular application [88]. Common textures used to modify the friction properties of a surface are micro dimples [89,90], micro stripes [91], micro grooves, and banded grooves [92]. The accuracy of feature definition, angle sharpness, edge angle, depth, and pitch all constitute factors that will influence the tribological properties. Surface wear needs to be considered for textured surfaces. However, it can be controlled through material selection and pattern design. For example, Aziz et al. used Fused Deposition Modeling (FDM) 3D printing to modify the tribological behavior of PLA surfaces [93].

### 7.8. Tool Surface Functionalization

The tool surface represents the boundary that controls the polymer/tool interactions. Tool topography can be modified to control polymer replication, achieving significant processing benefits. Different strategies have been used to achieve that, particularly surface modification using coatings and surface texturing [94,95,96,97]. The most significant benefits of surface engineering were observed for the filling of the micro- or nano-scale features and the ejection of the replicated texture from the tool. This section focuses on the effects of texturing on polymer flow and part ejection from the tool.

#### 7.8.1. Filling

Texturing has been demonstrated as an effective way to improve the filling of thin-wall injection molding cavities by reducing the required pressure drop [98]. Reducing the pressure requirement can lead to advantages for plastic product design, processing energy requirements, and resin selection. The effect of tool texturing on the polymer flow changes the rheological and thermal polymer/mold interface interactions.

**Figure 17:**
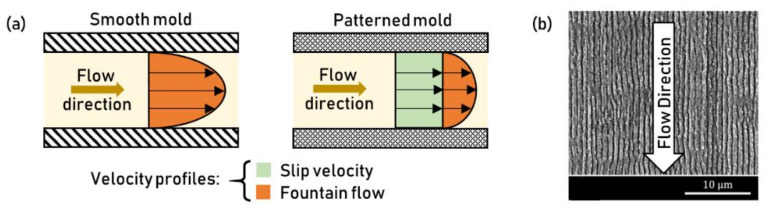
(**a**) Effect of wall slip on the polymer velocity profile in a thin-wall injection molding cavity; (**b**) ripples aligned along the polymer flow to facilitate wall slip.

Submicron scale textures realized using ultrafast pulsed laser have shown an effect on the onset of the wall slip phenomena (Figure 17). When touching the mold surface, the polymer macromolecules cannot reach the depth of the texture valleys, thus the absorption points are less than a flat surface. The viscosity, the molecular mass distribution, and the polymer chains’ relaxation time play an essential role in the phenomena. A polymer with high viscosity, narrow molecular mass distribution, and long relaxation time is more likely to slip [99].

Micro- and nano-scale texturing can also affect the filling flow by changing the thermal boundary condition at the polymer/mold interface [63,100]. The air pockets trapped in the troughs in the texture have low thermal conductivity, thus they delay the heat transfer to the mold and keep the polymer warmer during filling. However, this effect is relevant only for thin-wall plastic parts, specifically an experimental threshold of 150 μm was identified [101,102].

#### 7.8.2. Ejection

The topography highly affects the ejection of the polymer part from the textured tool. Several strategies have been adopted in the industry to decrease the force required to separate the polymer from the tool. In particular, the effect of surface roughness has been widely studied and exploited [103]. The force required to separate a plastic part from the mold generally increases with a rougher surface. However, highly polished tool surfaces also lead to high forces due to the vacuum generated at the interface. The optimization of mold topography through texturing has been demonstrated as an effective solution to solve demolding issues [104].

The understanding of ejection friction requires the analysis of the adhesion and deformation components [105]. The adhesion term is a surface effect related to chemical, electrostatic, and capillary adhesion. The deformation term is controlled by the mechanical interlocking generated between the polymer and the texture [106]. Surface engineering strategies control both components of ejection friction. In particular, mold surface generation using texturing controls the deformation component, while modification using coatings controls the adhesion [97].

## 8. Conclusions

**Figure 18:**
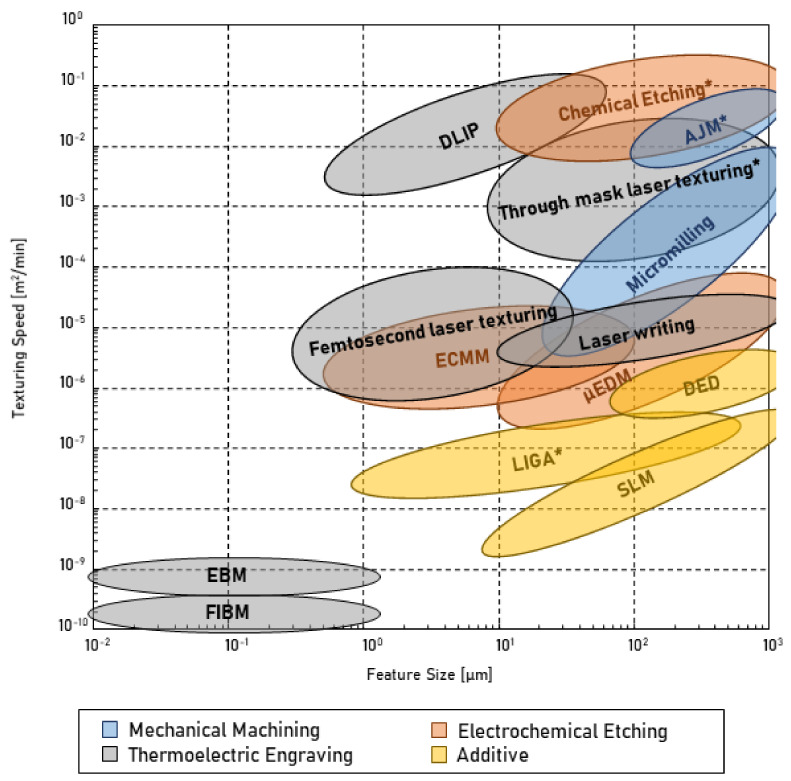
Feature size and texturing speed capabilities of surface texturing technologies. Multi-step technologies are indicated with a star (*).

The different texturing technologies reviewed in this work are summarized in Figure 18 as a function of the feature size and texturing speed. The characteristics of the technologies and the achievable textures can be summarized as follows:

- Chemical etching is the fastest technology and allows texturing of large surfaces. However, the structures that can be obtained are not very small and not very accurate. The most significant applications are for the automotive industry, which uses the technology for interior parts’ aesthetic texturing.
- The smallest texture can be manufactured using either EBM or FIBM. Both technologies are very accurate but extremely slow, limiting their diffusion to niece applications and small surfaces. The main applications, still limited to research labs and clean room environments, are high-end biomedical and electronics.
- Micro-milling is sensitive to the dimensions of the feature. The smaller is the texture, the slower is the process. Conventional micro-milling allows texturing down to 100 μm, while smaller textures (i.e., down to 20 μm) can be achieved using the ultrasound-assisted process. The most significant applications are headlight diffractors, for which sub-millimeter scale prisms are textured on injection mold surfaces.
- The μEDM texturing technology is susceptible to the required surface finish. Relatively smooth surfaces can be manufactured using low-spark energy while significantly decreasing the texturing speed. Texturing injection molds using this technology allows for overcoming the size limitations of micro-milling.
- The ECMM is faster than μEDM as it exploits the chemical energy to remove the material. Moreover, the more homogeneous material removal results in a better surface finish.
- When considering additive manufacturing technologies, DED is faster than SLM because the powder is deposited by the nozzle and sintered by the co-axial beam. However, SLM can manufacture smaller and more accurate texture features.
- DLIP is the fastest laser technology because it exploits the pattern interference to create features at specific locations on the surface. The functioning principles limit the type of textures that can be manufactured. However, the Through Mask Laser Texturing overcomes this limitation when considering textures with bigger feature sizes.
- Direct laser writing is a slow technology because of the direct material ablation, and the spot size dimension limits the feature size. Smaller textures, with limited geometries, can be realized using a femtosecond laser.
- The AJM texturing process is used for its high velocity and its cleanliness. However, it is not very accurate, and the feature definition is typically not high.
- The successful utilization of a texture for injection molding requires the evaluation of polymer processing aspects that are not discussed in this work; they are, however, well-established in the literature.

## Data Availability

Not applicable.
